# Studies on Novel Diagnostic and Predictive Biomarkers of Intrahepatic Cholestasis of Pregnancy Through Metabolomics and Proteomics

**DOI:** 10.3389/fimmu.2021.733225

**Published:** 2021-10-14

**Authors:** Ruirui Dong, Ningzhen Ye, Shaojie Zhao, Gaoying Wang, Yan Zhang, Tiejun Wang, Ping Zou, Jing Wang, Tingting Yao, Minjian Chen, Conghua Zhou, Ting Zhang, Liang Luo

**Affiliations:** ^1^ The Affiliated Wuxi Matemity and Child Health Care Hospital of Nanjing Medical University, Wuxi, China; ^2^ State Key Laboratory of Reproductive Medicine, Institute of Toxicology, School of Public Health, Nanjing Medical University, Nanjing, China; ^3^ Key Laboratory of Modern Toxicology of Ministry of Education, School of Public Health, Nanjing Medical University, Nanjing, China; ^4^ School of Computer Science and Telecommunication Engineering, Jiangsu University, Zhenjiang, China; ^5^ The Affiliated Wuxi No.2 People’s Hospital of Nanjing Medical University, Wuxi, China

**Keywords:** intrahepatic cholestasis of pregnancy, omics, Acyl-CoA oxidase 1, L-palmitoylcarnitine, glycocholic acid

## Abstract

**Background:**

Intrahepatic cholestasis of pregnancy (ICP) usually occurs in the third trimester and is associated with increased risks in fetal complications. Currently, the exact mechanism of this disease is unknown. The purpose of this study was to develop potential biomarkers for the diagnosis and prediction of ICP.

**Methods:**

We enrolled 40 pregnant women diagnosed with ICP and 40 healthy pregnant controls. The number of placental samples and serum samples between the two groups was 10 and 40 respectively. Ultra-performance liquid chromatography tandem high-resolution mass spectrometry was used to analyze placental metabolomics. Then, we verified the differentially expressed proteins and metabolites, both placental and blood serum, in the first, second, and third trimesters.

**Results:**

Metabolomic analysis of placental tissue revealed that fatty acid metabolism and primary bile acid biosynthesis were enriched. In the integrated proteomic and metabolomic analysis of placental tissue, peroxisomal acyl-CoA oxidase 1 (ACOX1), L-palmitoylcarnitine, and glycocholic acid were found to be three potential biomarkers. In a follow–up analysis, expression levels of both placental and serum ACOX1, L-palmitoylcarnitine, and glycocholic acid in both placenta and serum were found to be significantly higher in third-trimester ICP patients; the areas under the ROC curves were 0.823, 0.896, and 0.985, respectively. Expression levels of serum ACOX1, L-palmitoylcarnitine, and glycocholic acid were also significantly higher in first- and second-trimester ICP patients; the areas under the ROC curves were 0.726, 0.657, and 0.686 in the first trimester and 0.718, 0.727, and 0.670 in the second trimester, respectively. Together, levels of the three aforementioned biomarkers increased the value for diagnosing and predicting ICP (AUC: 0.993 for the third, 0.891 for the second, and 0.932 for the first trimesters).

**Conclusions:**

L-palmitoylcarnitine, ACOX1, and glycocholic acid levels taken together may serve as a new biomarker set for the diagnosis and prediction of ICP.

## Introduction

Intrahepatic cholestasis of pregnancy (ICP) is a common and severe disease that occurs in the third trimester of pregnancy. The incidence of ICP ranges from 0.1- 2% (1); the condition is always accompanied by pruritus, elevated hepatic liver enzymes, and elevated serum total bile acid (TBA) levels (2). In recent 20 years, ursodeoxycholic acid (UDCA) and S-adenosylmethionine (SAMe) have demonstrated in clinical trials and observational studies a beneficial effect on pruritus and serum biochemical abnormalities, further improving perinatal outcome (3). Although ICP is rarely harmful to the mother, premature delivery, amniotic fluid contamination, fetal distress, and even fetal death are severe and common complications. Therefore, early prediction and diagnosis can provide better prevention and treatment of ICP (4). The etiology and pathogenesis of ICP, however, remain unclear (5). Several studies have reported inflammation, apoptosis, oxidative stress, lipid metabolism, cell growth, and the immune response to all be closely related to the manifestation of ICP (6). Levels of TBA remain the most important laboratory index for ICP diagnosis, although TBA elevation also occurs in patients suffering a variety of hepatobiliary illnesses such as hepatolithiasis, viral and autoimmune hepatitis, and acute fatty liver disease of pregnancy (7, 8). Thus, the use of serum levels of TBA to diagnose ICP has certain limitations. New diagnostic and prognostic ICP biomarkers are urgently required.

Multi-omics techniques offer the potential to comprehensively analyze molecules that compose cells, tissues, and organs as a means for collectively understanding complex systems (9). These techniques allow for the study of genes, mRNA, proteins, and metabolites, which are not only useful in understanding basic physiological processes, but may also contribute to diagnostic screening (10, 11). Integrative omics techniques have not been previously applied in the study of ICP.

In our previous study, we used iTRAQ analysis combined with liquid chromatography-tandem mass spectrometry (LC-MS/MS) to separate differentially expressed placental proteins from four ICP patients and four healthy pregnant women (12). Here, ultra performance LC-tandem high resolution MS was used to comparatively analyze the placental metabolomics of pregnant women suffering ICP and of healthy pregnant women. In the follow-up study, we verified the expression of differentially expressed serum and placental proteins and metabolites. The objective of this study was to elucidate ICP-specific or abnormally expressed proteins and metabolites that could serve as biomarkers for the prediction and diagnosis of ICP.

## Materials and Methods

### Patient Recruitment and Sample Collection

We enrolled 40 pregnant women diagnosed with ICP and 40 healthy pregnant controls receiving care at the Affiliated Wuxi Maternity and Child Health Care Hospital of Nanjing Medical University from October 2017 to October 2020. The 10 study participants who provided placental samples also provided serum samples. Placental tissue samples used in this study were randomly collected from 10 women with uncomplicated pregnancies and 10 women with pregnancies complicated by ICP. In order to be consistent with the number of samples used in previous proteomics studies, we selected 4 placental samples from each group for preliminary metabolomics analysis. At the same time, 10 placental tissues were selected for validation of metabolites in each group.

All subjects were primiparous Chinese women with singleton pregnancies. Women presenting with classical pruritus associated with liver dysfunction and raised serum bile acids (all of which subsequently resolved after delivery) were diagnosed with ICP. The diagnostic criteria of ICP as well as both inclusion and exclusion criteria have previously been described (4). Briefly, patients suffering from other causes of liver dysfunction, including preeclampsia, hemolysis, elevated liver enzymes and low platelets (HELLP) syndrome, acute fatty liver of pregnancy, primary biliary cirrhosis, viral hepatitis, and any ultrasound abnormalities that could have resulted in biliary obstruction were excluded from analysis. Placentas were collected from both ICP patients as well as healthy controls after cesarean section according to a protocol previously published (13). Briefly, five punch biopsies from various areas of each placenta were randomly pooled immediately after caesarian section and washed in cold normal saline to eliminate any contaminating blood. Placental tissue was then immediately frozen in liquid nitrogen for proteomics and metabolomics analyses as well as western blotting. For immunohistochemical analysis, placental tissue was fixed in PBS with 10% formalin for 24 h at 4°C, then dehydrated in a graded series of ethanol and embedded in paraffin.

Serum samples taken during the third trimester (<28 weeks) were collected directly from 40 pregnant women diagnosed with ICP and 40 healthy pregnant controls. At the same time, the serum samples of the first (8-14 weeks) and second (16-20 weeks) trimesters of all pregnant women in a repository were collected retrospectively. However, due to the low amount of serum stored, 24 and 20 serum samples were used to detect L-palmitoylcarnitine in first- and second-trimester ICP patients. The serum samples used to detect L-palmitoylcarnitine in the control group were 40 and 33 in first- and second-trimester. Venous blood samples (5 mL) were collected from all participants and isolated within 4 h by centrifugation at 4,000 rpm for 10 min. Serum samples were stored at -80°C prior to analyses. No patient underwent ursodeoxycholic acid treatment prior to blood sample collection. Ethics approval was granted by the Institutional Review Board of Nanjing Medical University, and all participants provided written, informed consent (Nanjing Medical University Ethical review (2016) 241 number). Patient characteristics, liver function test data and perinatal outcomes are summarized in **Table 1**.

**Table 1:** Clinical characteristics and perinatal outcomes of pregnant women with ICP and healthy pregnant controls.

Variable	ICP (mean±SD)	Control (mean±SD)	P-value
**Placental samples (n)**	10	10	/
**Serum samples (n)**	40	40	/
**Maternal age (years)**	28.8 ± 5.3	27.9 ± 3.0	0.272
**Gestational weeks** **(Blood collection)**	36.9 ± 1.9	36.3 ± 0.4	0.064
**TBA (μmol/L)**	32.4 ± 18.0	3.5 ± 1.8	0.000
**ALT (IU/L)**	80.6 ± 114.2	10.9 ± 5.8	0.000
**AST (IU/L)**	66.5 ± 82.7	18.1 ± 14.0	0.001
**Gestational weeks** ** (Delivery)**	38.6 ± 1.8	39.7 ± 0.6	0.000
**Newborn weight (g)**	3176.9 ± 648.5	3348.3 ± 503.2	0.295

TBA, total bile acid; ALT, alanine transaminase; AST, aspartate transaminase. Statistical analyses were performed using the t-test; *P < 0.05 was considered to reflect significance.

### Proteomics

Proteomics analysis was performed as previously described by us (11). Briefly, frozen placental tissue was dissolved in a lysis buffer containing 7 M urea, 2 M thiourea, 2%(w/v) DTT and 1% (v/w) protease inhibitor cocktail at 4°C for 1 h. The supernatant was collected and protein concentration determined according to the Bradford method using bovine serum albumin (BSA) as the standard. Trypsin digestion and iTRAQ labeling were performed according to manufacturer instructions (Applied Biosystems, Foster City, CA, USA).

Labeled peptide mixtures were resuspended in SCX chromatography Buffer A (10 mM NH4COOH, 5% ACN, pH 2.7) and loaded onto a strong cation-exchange column (1 mm ID * 10 cm packed with Poros 10S; DIONEX, Sunnyvale, CA, USA) for fractionation. Effluents were monitored at 214 nm based on the UV light trace and fractions were collected every 2 min; a total of 20 fractions were obtained.

The 20 fractions were sequentially loaded onto a μ-precolumn^TM^ cartridge (0.3 * 5 mm, 5 μm, 100 A; DIONEX) at a flow rate of 20 μl/min. Trap column effluent was then transferred to a reverse-phase microcapillary column (0.0756 * 150 mm, Acclaim PepMap100 C18 column, 3 μm, 100 A; DIONEX). Peptide analysis was performed using an LTQ Orbitrap Velos (Thermo Fisher Scientific, San Jose, CA, USA) coupled directly to an LC column.

Peak lists were generated using MS machine software. Raw files were used to search the International Protein Index (IPI) human proteome database (version 3.83; 93289 sequences) (14) using MaxQuant (version 1.2.2.5) (15). Protein quantification was calculated by combining MaxQuant identification results with the local Libra quantification algorithm (16), except that quantification signals were extracted from corresponding HCD-MS3 spectra. For the identification of differentially expressed proteins, cutoffs for fold change and P value (Student’s t-test) were set to 1.5 and 0.05, respectively.

An analysis of cellular processes influenced by differentially expressed proteins from both women suffering ICP and healthy pregnant women was performed using Pathway Studio (v7.00) software (Ariadne Genomics, Inc., Rockville, MD, USA) (17). Cellular processes influenced by differentially expressed proteins were determined by searching the database for imported genes/proteins and for cellular processes in which the imported genes/proteins are involved. Each identified cellular process was confirmed manually using the relevant PubMed/Medline hyperlinked abstracts. The mass spectrometry proteomics data have been deposited to the ProteomeXchange Consortium via the PRIDE (18) partner repository with the dataset identifier PXD028734.

### Metabolomics

Metabolomic analysis was performed as previously described by us (19). Briefly, placental tissue samples (50 mg) were mixed with water and methanol (volume ratio 1:3) and vortex-mixed for 30 sec. The mixture was centrifuged at 16,000 g for 15 min at 4°C to precipitate proteins. Next, the supernatant was dried using a centrifugal concentration dryer (Labconco, Kansas City, MO, USA) and reconstituted for ultra-performance LC-Q-Exactive analyses.

Samples were analyzed using the UltiMate3000 high-performance LC system (Dionex, Germering, Germany) coupled with the Q-Exactive MS machine (Thermo Fisher Scientific, Bremen, Germany). Chromatographic separation was performed using a Hypersil GOLD C18 column (100 mm × 2.1 mm, 1.9 μm; Thermo Fisher Scientific). The mobile phase A was a solution of acetonitrile containing 0.1% formic acid, and the mobile phase B was a solution of water containing 0.1% formic acid. The flow rate was 0.4 mL/min and the column temperature was kept at 40 °C. An optimal linear gradient program was shown as follows: 0–3 min, 1% A; 3–10 min, 1%–99% A; 10–13 min, 99% A; 13–13.1 min, 99–1% A; 13.1–15 min, 1% A.

Mass data acquisition was performed using the Q-Exactive MS machine with a heated electrospray ionization (HESI) source operating in positive and negative ion modes. (The use of both positive and negative ionization data generally increases the coverage of metabolites and information abundance of metabolomics analysis. However, when both positive and negative ionizations were detected, it limited the optimization of the detection sensitivity of specific metabolites suitable for a specific ionization mode.) The capillary voltage was 3500 V for the positive ion mode, and 2500 V for the negative ion mode. The capillary temperature was 250°C and the heater temperature was 425°C for both modes. A sheath gas flow of 50 arbitrary units (AU), an auxiliary gas flow of 13 AU, and a sweep gas flow of 0 AU were utilized during analyses. The S-Lens RF level was 60. Data acquisition was performed in a full-scan mode ranging from 70-1050 m/z and the resolution was 70000. Metabolite identification was based on the comparison of accurate mass and retention time with metabolite standards using TraceFinder 3.1 (Thermo Fisher Scientific). All samples were analyzed in a randomized fashion. The QC samples and internal standards were used and monitored for the quality control of the analysis, which verified the validity of the metabolomics results.

To further characterize relevant metabolic changes and metabolic pathways, differentiated metabolites were first annotated using the Kyoto Encyclopedia of Genes and Genomes (KEGG, http://www.genome.jp/kegg/) and Human Metabolome Database (HMDB, http://www.hmdb.ca/). Both databases were accessed on April 19, 2018. Data were subsequently processed and analyzed using MetaboAnalyst 4.0 (http://www. Metaboanalyst.ca/MetaboAnalyst/) and R software (v3.4.3, GitHub). The pathway and enrichment analysis MetaboAnalyst modules, which were based on KEGG and Small Molecule Pathway (SMPdB, http://smpdb.ca/) databases, respectively, were used (20).

### Western Blotting

Frozen tissues from both groups were dissolved in a RIPA lysis buffer containing 50mM Tris(pH 7.4), 150mM NaCl, 1% NP-40, 0.5% sodium deoxycholate and 1% (v/w) protease inhibitor cocktail (PMSF) for protein extraction as described above. Samples containing 100 mg of protein from ICP patient and normal control placental tissue were electrophoresed on a 12% SDS polyacrylamide gel and transferred to a nitrocellulose membrane (GE Healthcare, San Francisco, CA, USA). Membranes were blocked in Tris-buffered saline (TBS) containing 5% non-fat milk powder for 1 h and incubated overnight with anti-ACOX1 (ab184032, 1:1000; Abcam, Cambridge, MA, USA) antibodies diluted in TBS/5% non-fat milk powder. Tubulin was used as a loading control. Membranes were washed three times (10 min each) with TBS and incubated for 1 h with horseradish peroxidase (HRP)-conjugated goat anti-rabbit IgG (1:1000; Beijing ZhongShan Biotechnology, Beijing, China). Specific proteins were detected using an ECL kit and AlphaImager (FluorChem 5500; ProteinSimple, San Jose, CA, USA). Protein expression levels were analyzed using AlphaEaseFC software (ProteinSimple).

### Immunohistochemistry

Formalin-fixed tissues from both ICP and control groups were embedded in paraffin, sectioned at 5 μm, and mounted on silane-coated slides. Sections were de-waxed and rehydrated by descending grades of alcohol and finally distilled water; endogenous peroxidase was blocked using 3% (v/v) hydrogen peroxidase in phosphate buffered saline (PBS). The sections were subjected to microwave antigen retrieval in 0.02 M EDTA, washed in PBS, and blocked with goat serum (Beijing ZhongShan Biotechnology) for 2 h. Sections were subsequently incubated overnight at 4°C with anti-ACOX1 (1:250). Sections were incubated with HRP-conjugated secondary antibody (1:1,000; Beijing ZhongShan Biotechnology) for 1 h at room temperature after being washed three times in PBS. Immunoreactivity was demonstrated using diaminobenzidine (Beijing ZhongShan Biotechnology) for increased sensitivity, which produced a brown insoluble precipitate at immunopositive sites. Sections were counterstained with hematoxylin and mounted with a cover glass. Negative controls were incubated with a solution devoid of primary antibodies. All immunostained sections were evaluated in a blinded manner by two observers. At the same time, Image J software was used to determine the intensity of staining in various parts of the placental sections from ICP and control groups.

### Validation of Differential Metabolite and Protein Expression

Serum and placental L-palmitoylcarnitine levels were detected using the ultra-performance LC Ultimate 3000 system coupled to a Q-Exactive MS machine. Placental glycocholic acid was detected using the Ultimate 3000 system coupled to a Q-Exactive MS machine. Samples were prepared as previously described by us (19). Conditions under which LC and MS were performed were as aforementioned in metabolomic profiling. Serum glycocholic acid levels were determined using a Beckman AU5821 autoanalyzer at the Clinical Laboratory Department of the Affiliated Wuxi Maternity and Child Healthcare Hospital of Nanjing Medical University. Serum ACOX1levels were determined using ELISA (BST Biological, China) according to manufacturer’s instructions.

### Statistical Analyses

All statistical analyses were performed using SPSS v. 22.0 (IBM, Armonk, NY, USA) and GraphPad Prism software. When the raw data were not suitable for t-test, we used log transformation of raw data to make them conform to normality (21). The data were normally distributed. Student’s t-test was used to compare demographic and clinical characteristics and biomarker expression levels among ICP and control groups. Receiver operator characteristic (ROC) curves for biomarker levels were constructed to derive sensitivities and specificities [with 95% confidence intervals (CIs)] by referencing areas under the curves (AUCs); an AUC greater than 0.70 was considered an acceptable level of discrimination. Pearson correlations between differentially expressed biomarker levels and serum TBA levels were also calculated. All results were expressed as means ± standard errors (SEs). A P-value <0.05 was considered to reflect statistical significance.

## Results

### Clinical Characteristics and Perinatal Outcomes of Pregnant Women With ICP and Healthy Pregnant Controls

Clinical characteristics and perinatal outcomes of validation samples are summarized in **Table 1**. We found no significant difference among the two groups in terms of maternal age, gestational age at the time of blood collection and newborn weight (both P *>*0.05). However, levels of TBA, alanine transaminase (ALT), and aspartate transaminase (AST) were significantly higher in pregnant women with ICP than in healthy pregnant controls (**Table 1**). Gestational age at the time of delivery were significantly lower in pregnant women with ICP than in healthy pregnant controls (**Table 1**). Clinical characteristics of screening samples can be found in our previous proteomics study of ICP using placental tissue (12).

### Placental Metabolomic Profile

**Figure 1:**
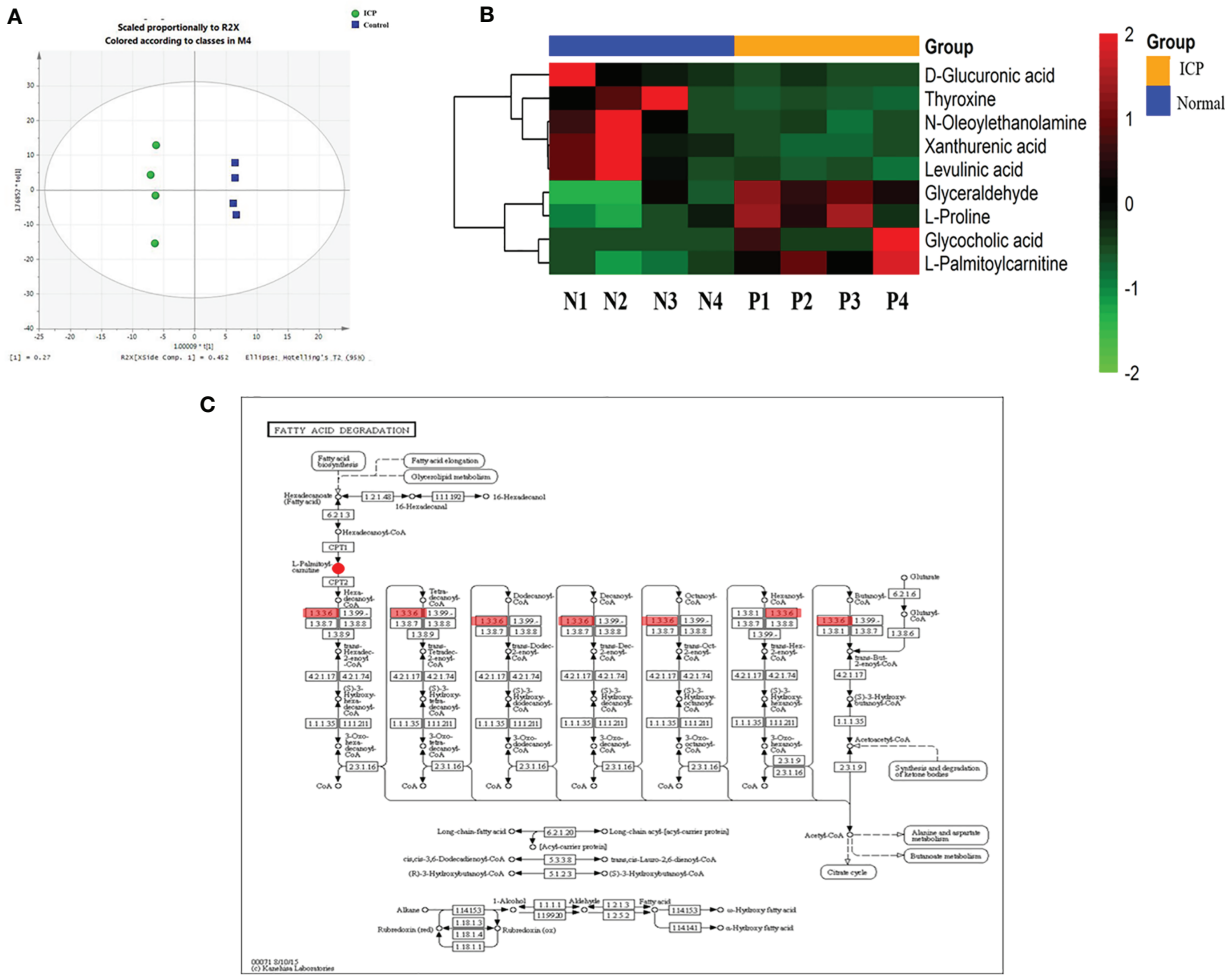
Integrated proteomics and metabolomics analysis. **(A)** OPLS-DA score plots derived from analysis of placental tissue by ultra-performance LC-Q-TOF/MS in positive and negative ionization modes. R^2^X and R^2^Y indicate the fraction of the variables explained by the model, while Q^2^ shows the predictive abilities of the model. R^2^X = 0.452, R^2^Y = 0.998, and Q^2^ = 0.803. The green circle is labeled as ICP; the blue square is labeled as control; **(B)** Cluster analyses of differentially expressed placental metabolites among pregnant women with ICP (P) and healthy pregnant women (N). Hierarchical cluster analyses of the nine differentially expressed metabolites revealed significantly altered levels in ICP patients. Metabolite levels are shown as colored boxes; red, high level; green, low level; **(C)** Part of the fatty acid metabolism pathway. The 1.3.3.6 are labeled as ACOX1. ACOX1 maintains a regulatory relationship with L-palmitoylcarnitine.

Placental metabolites of pregnant women with ICP and healthy pregnant controls were studied; pretreated data were imported into SIMCA 13.0 for multivariate statistical analysis. Supervised data analysis was carried out by applying OPLS-DA to obtain the OPLS-DA scores of collected data in both positive and negative ion modes (**Figure 1A**). Results of the OPLS-DA analysis revealed that placental metabolites of pregnant women with ICP significantly differed from those of the controls. The R^2^Y and Q^2^ values of the OPLS-DA model were 99.8% and 80.3%, respectively, indicating that the two groups were well-distinguishable.

### Identification of Differential Metabolites

Ultimately, a total of 178 metabolites in placental tissue of ICP patients were annotated from detected spectral features obtained using the ultra-performance LC Q-Exactive machine. Potential metabolites were selected based on the variable important for the projection (VIP) value (>1) and P value (<0.05). A total of 9 metabolites meeting these criteria were identified. Among the different metabolites, four exhibited a remarkable increase in relative intensities while five were found to be decreased in ICP patients as compared to controls (**Table 2**). Cluster analyses were used to define specific expression patterns of all nine metabolites; expression levels significantly differed among pregnant women with ICP and healthy pregnant controls (**Figure 1B**).

**Table 2:** Differentially expressed placental metabolites among pregnant women with ICP and healthy pregnant women.

Metabolites	VIP	Fold change (P:C)	P-value	Change in expression level
**Glyceraldehyde**	1.60	1.56	1.05E-02	UP
**L-Proline**	1.48	1.78	2.47E-02	UP
**Glycocholic acid**	**1.69**	**80.72**	**0.43E-02**	**UP**
**L-Palmitoylcarnitine**	**1.58**	**2.71**	**0.65E-02**	**UP**
**D-Glucuronic acid**	1.44	0.17	3.25E-02	DOWN
**Thyroxine**	1.33	0.20	1.71E-02	DOWN
**Xanthurenic acid**	1.36	0.31	1.14E-02	DOWN
**Levulinic acid**	1.27	0.25	4.35E-02	DOWN
**N-Oleoylethanolamine**	1.20	0.32	4.82E-02	DOWN

Potential biomarkers were extracted based on variable important for the projection (VIP) >1. Table contains data for metabolites that were ≥1.5-fold upregulated or ≤0.67-fold downregulated in pregnant women with ICP (P) compared to healthy pregnant women (C). The P : C ratios are shown. The key metabolites data are bolded.

### Metabolic Pathway Analysis

The nine ICP-associated metabolites were input into MetaboAnalyst for analysis; a summary of the pathway analysis is shown in **Table 3**. The predominant metabolites were found to be those involved in fatty acid metabolism, tyrosine metabolism, inositol phosphate metabolism, ascorbate and aldarate metabolism, primary bile acid biosynthesis, starch and sucrose metabolism, pentose and glucuronate interconversion, aminoacyl-tRNA biosynthesis, arginine and proline metabolism, tryptophan metabolism, and amino sugar and nucleotide sugar metabolism. Fatty acid metabolism and primary bile acid biosynthesis were found to be significantly associated with ICP.

**Table 3:** Pathway analysis of metabolite changes in ICP in the placenta.

Pathway Name	Total	P-value
**Fatty acid metabolism**	**50**	**2.51E-02**
**Primary bile acid biosynthesis**	**47**	**4.06E-02**
**Inositol phosphate metabolism**	39	1.29E-01
**Ascorbate and aldarate metabolism**	45	8.92E-02
**tyrosine metabolism**	76	2.02E-01
**Starch and sucrose metabolism**	50	4.29E-01
**Pentose and glucuronate interconversions**	53	4.44E-01
**Aminoacyl-tRNA biosynthesis**	75	6.78E-01
**Arginine and proline metabolism**	77	5.74E-01
**Tryptophan metabolism**	79	3.79E-01
**Amino sugar and nucleotide sugar metabolism**	88	5.61E-01

The analysis was conducted by the module of enrichment analysis of MetaboAnalyst 4.0. *P<0.05 was considered to reflect significance. The key metabolic pathway are bolded.

### Integrated Proteomics and Metabolomics Analysis

Results of our proteomics analysis of placental tissue from both ICP and control group women can be found in *PLOS ONE* (12). Based on the KEGG analysis, a shared pathway of metabolites and differentially abundant proteins was found (**Figure 1C**). Findings indicated that fatty acid metabolism plays an important role in the pathogenesis of ICP. Peroxisomal ACOX1 was found to participate in a regulatory relationship with L-palmitoylcarnitine (**Figure 1C**). ACOX1 was the differentially expressed protein while L-palmitoylcarnitine was the differentially expressed metabolite. Thus, we selected both a protein and a metabolite as potential biomarkers for validation.

### Western Blot and Immunohistochemical Protein Analysis

**Figure 2:**
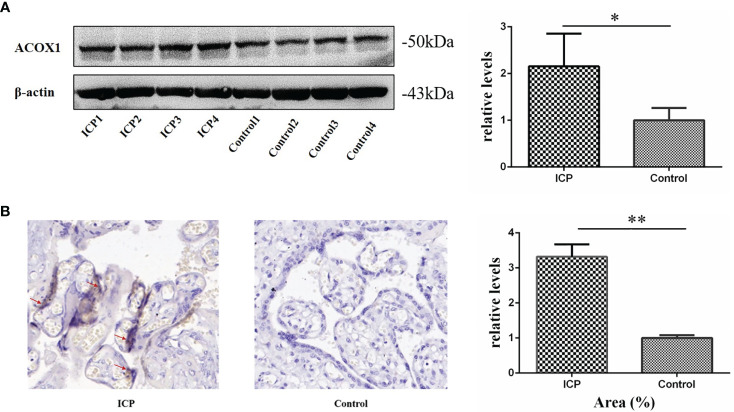
Validation of ACOX1 protein in placental tissue. **(A)** Western blot analysis of placental ACOX1 in tissue obtained from pregnant women with ICP and healthy pregnant women. Placental ACOX1 levels were significantly increased among pregnant women with ICP as compared to healthy pregnant women (P=0.015); β-actin was used as an internal control. (*P < 0.05); **(B)** Immunohistochemical staining for placental ACOX1 among 4 pregnant women with ICP and 4 healthy pregnant women (×400). Immunohistochemistry revealed higher cytoplasmic and nuclear levels of ACOX1 in placental trophoblasts of pregnant women with ICP as compared to healthy pregnant women. The mean positive signal intensity of placenta in ICP group was significantly higher than that in control group (**P < 0.01).

Western blot and immunohistochemistry validated our proteomic findings and further verified the roles of ACOX1 in ICP pathogenesis. Expression levels of ACOX1, as quantified via western blotting, were found to be significantly increased in placental samples obtained from ICP patients as compared to control group tissues (**Figure 2A**; P <0.05). Immunohistochemical analysis was performed to define the intracellular location of ACOX1 in human placental cells; ACOX1 was found to be expressed predominantly in the cytoplasm and/or nucleus of syncytiotrophoblasts and cytotrophoblasts (**Figure 2B**). The mean positive signal intensity of placenta in ICP group was significantly higher than that in control group (**Figure 2B**; P <0.01). Expression patterns of ACOX1 were consistent with proteomics and western blot analyses.

### Validation of Differential Biomarker Expression Levels in the Third Trimester

**Figure 3:**
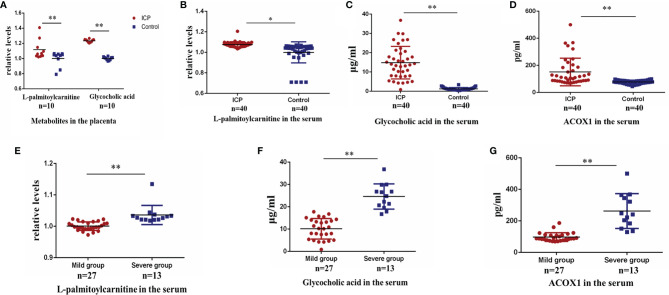
Validation of alteration in protein and metabolite levels in late pregnancy. **(A)** Placental levels of L-palmitoylcarnitine and glycocholic acid in ICP and control groups (P=0.006, P=0.000, respectively); **(B)** serum levels of L-palmitoylcarnitine in ICP and control groups (P=0.010); **(C)** serum levels of glycocholic acid in ICP and control groups (P=0.000); **(D)** serum levels of ACOX1 in ICP and control groups (P=0.000). **(E)** serum levels of L-palmitoylcarnitine in mild and severe ICP groups (P=0.000); **(F)** serum levels of glycocholic acid in mild and severe ICP groups (P=0.000); **(G)** serum levels of ACOX1 in mild and severe ICP groups (P=0.000). (**P < 0.01; *P < 0.05).

The L-palmitoylcarnitine data (serum and placenta) and the glycocholic acid data (placenta) were log transformed for further analysis. Levels of placental L-palmitoylcarnitine and glycocholic acid were found to be significantly elevated in pregnant women with ICP as compared to controls (P<0.01; **Figure 3A**). Serum levels of L-palmitoylcarnitine and glycocholic acid were also found to be significantly elevated in ICP group patients as compared to controls (P<0.05, P<0.01, respectively; **Figures 3B, C**). The differentially expressed protein ACOX1 was found to be significantly upregulated in the serum of ICP patients as compared to controls (P<0.01; **Figure 3D**). At the same time, ICP patients were divided into mild group (TBA<40μmol/L) and severe group (TBA≥40μmol/L). Serum levels of L-palmitoylcarnitine, glycocholic acid and ACOX1 were also found to be significantly elevated in severe group as compared to mild group (P<0.01, **Figures 3E**–**G**).

### Validation of Differential Biomarker Expression Levels in First and Second Trimesters

**Figure 4:**
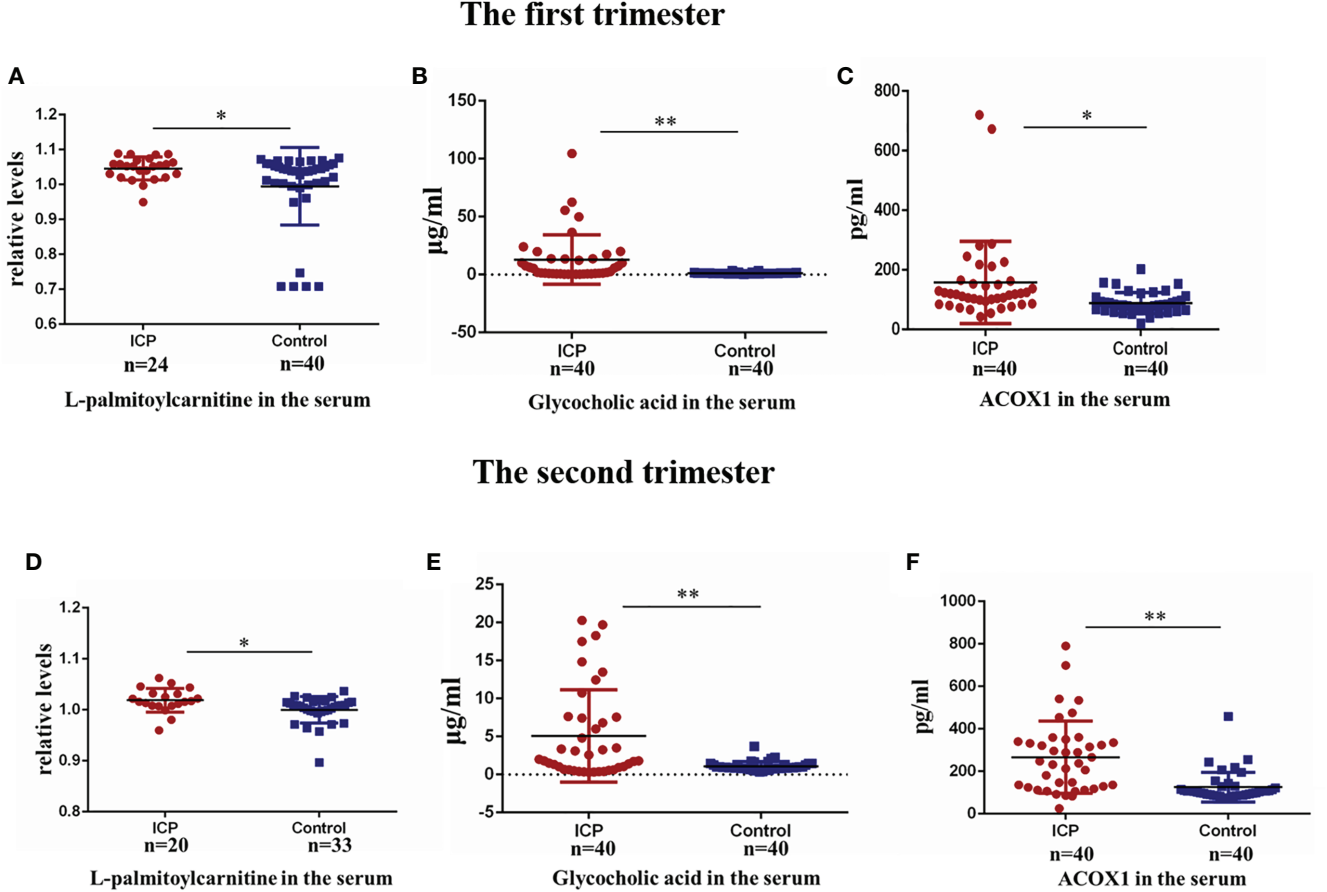
Validation of alteration in biomarker levels in the first and second trimester. **(A)** Serum levels of L-palmitoylcarnitine in the first trimester(P=0.040); **(B)** serum levels of glycocholic acid in the first trimester (P=0.000); **(C)** serum levels of ACOX1 in the first trimester (P=0.022); **(D)** Serum levels of L-palmitoylcarnitine in the second trimester(P=0.012); **(E)** serum levels of glycocholic acid in the second trimester (P=0.000); **(F)** serum levels of ACOX1 in the second trimester (P=0.000). (*P < 0.05; **P < 0.01).

The L-palmitoylcarnitine data were log transformed for further analysis. Serum levels of L-palmitoylcarnitine, glycocholic acid, and ACOX1 were found to be significantly upregulated in ICP patients as compared to controls in early pregnancy (P<0.05, P<0.01, and P<0.05, respectively; **Figures 4A**–**C**). Serum levels of these compounds were found to also be significantly elevated in ICP patients as compared to controls during the second trimester (P<0.05, P<0.01, and P<0.01, respectively; **Figures 4D**–**F**).

### Diagnostic Utility of Third-Trimester Serum Differential Biomarker Levels

**Figure 5:**
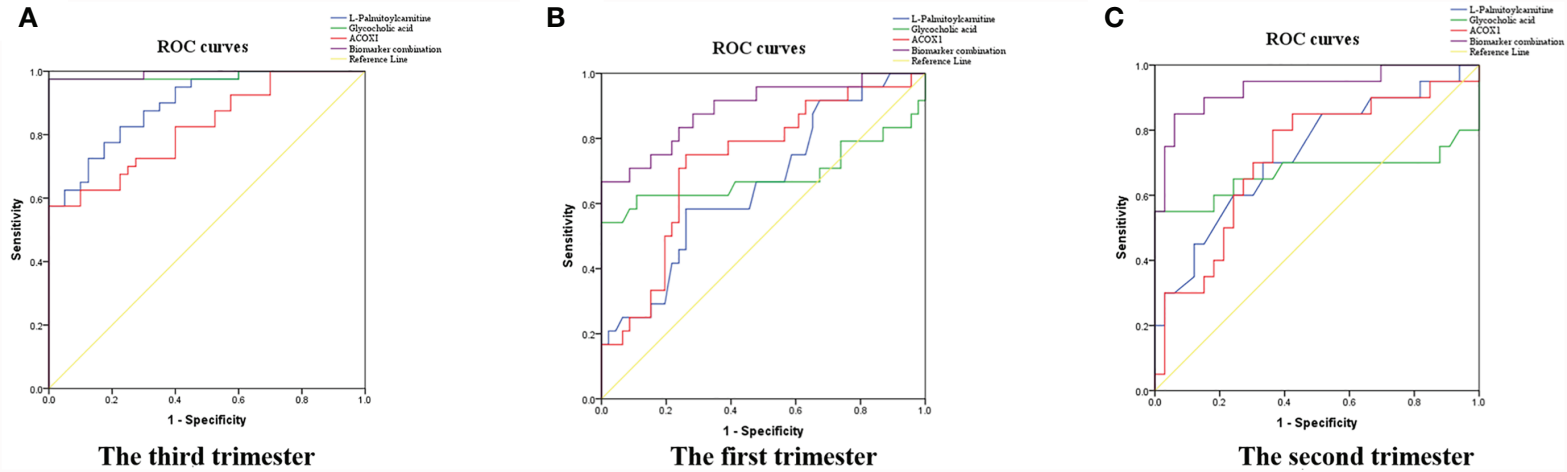
Diagnostic and predictive utility of maternal serum biomarker levels among pregnant women with ICP. **(A)** Diagnostic value in the third trimester; **(B)** predictive value in the first trimester; **(C)** predictive value in the second trimester; the three biomarkers combined resulted in the greatest AUC.

To evaluate the sensitivities and specificities of metabolite and protein signatures in terms of ICP diagnosis, ROC curves were constructed and AUCs calculated (**Figure 5A**); these were 0.896, 0.985, and 0.823 for L-palmitoylcarnitine, glycocholic acid, and ACOX1, respectively (**Figure 5A**). We assessed the diagnostic utility of L-palmitoylcarnitine, glycocholic acid, and ACOX1 at cutoff values of 8.08, 3.83, and 99.84, respectively (**Table 4**); the Youden index confirmed that these cutoffs were optimal. Multiple logistic regression analyses of combined biomarker data yielded an AUC of 0.993 (**Table 4**). Thus, the metabolites and proteins combined into a single biomarker improved the diagnostic value of the compounds.

**Table 4:** Diagnostic utility of serum biomarker levels among pregnant women with ICP in the third trimester.

Biomarker	AUC	95%CI	Cutoff Value
**L-Palmitoylcarnitine (log)**	0.896	0.831-0.961	8.08
**Glycocholic acid (μg/ml)**	0.985	0.955-1.000	3.83
**ACOX1(pg/ml)**	0.823	0.733-0.913	99.84
**Biomarker combination**	0.993	0.977-1.000	/

**Figure 6:**
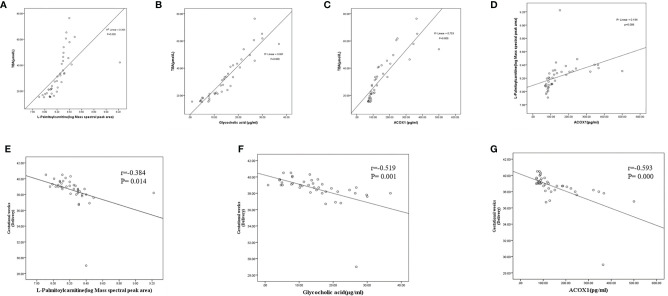
Correlations between serum biomarker levels and gestational age (delivery) among pregnant women with ICP in the third trimester. **(A)** L-palmitoylcarnitine and TBA (r=0.395, P=0.000); **(B)** glycocholic acid and TBA (r=0.861, P=0.000); **(C)** ACOX1 and TBA (r=0.753, P=0.000); **(D)** ACOX1 and L-palmitoylcarnitine (R^2^=0.154, P=0.006); **(E)** L-palmitoylcarnitine and gestational weeks (delivery) (r=-0.384, P=0.014); **(F)** glycocholic acid and gestational weeks (delivery) (r=-0.519, P=0.001); **(G)** ACOX1 and gestational weeks (delivery) (r=-0.593, P=0.000).

At the same time, we calculated Pearson correlations between levels of TBA and L-palmitoylcarnitine, glycocholic acid, and ACOX1. Levels of all three biomarkers were found to be positively associated with TBA levels (r=0.395, P<0.01; r=0.861, P<0.01; and r=0.753, P<0.01 for L-palmitoylcarnitine, glycocholic acid, and ACOX1, respectively; **Figures 6A**–**C**). Calculation of Pearson correlations between ACOX1 and L-palmitoylcarnitine levels revealed L-palmitoylcarnitine to be positively correlated with ACOX1 levels (r=0.184, P<0.01; **Figure 6D**). At the same time, we calculated Pearson correlations between levels of gestational age (delivery) and L-palmitoylcarnitine, glycocholic acid, and ACOX1. Levels of all three biomarkers were found to be negatively associated with gestational age (delivery) levels (r=-0.384, P<0.05; r=-0.519, P<0.01; and r=-0.593, P<0.01 for L-palmitoylcarnitine, glycocholic acid, and ACOX1, respectively; **Figures 6E**–**G**).

### Predictive Utility of Serum Differential Biomarker Levels in the First and Second Trimesters

To evaluate the sensitivities and specificities of metabolite and protein signatures regarding ICP prediction, ROC curves were constructed and AUCs calculated for first and second trimester samples (**Figures 5B, C**); these were 0.657, 0.686, and 0.726 for L-palmitoylcarnitine, glycocholic acid, and ACOX1 in early pregnancy (**Figure 5B**) and 0.727, 0.670, and 0.718 in the second trimester (**Figure 5C**). We assessed the predictive utility of L-palmitoylcarnitine, glycocholic acid, and ACOX1 at cutoff values of 8.06, 5.18, and 94.39 in early pregnancy and at cutoff values of 7.93, 2.77, and 111.37 in the second trimester (**Table 5**); the Youden index indicated that these cutoffs were optimal. Multiple logistic regression analyses of the combined biomarker data yielded an AUC of 0.891 and 0.932 for early pregnancy and the second trimester, respectively (**Table 5**). A combination of metabolites and proteins was thus found to improve the value of the aforementioned compounds in the context of ICP prediction.

**Table 5:** Predictive utility of serum biomarker levels among pregnant women with ICP in the first and second trimesters.

Biomarker	The first trimester			The second trimester		
	AUC	95%CI	Cutoff Value	AUC	95%CI	Cutoff Value
**L-Palmitoylcarnitine (log)**	0.657	0.522-0.792	8.06	0.727	0.583-0.871	7.93
**Glycocholic acid (μg/ml)**	0.686	0.520-0.851	5.18	0.670	0.480-0.860	2.77
**ACOX1(pg/ml)**	0.726	0.599-0.852	94.39	0.718	0.571-0.865	111.37
**Biomarker combination**	0.891	0.806-0.977	/	0.932	0.855-1.000	/

## Discussion

Studies have shown the perinatal mortality of ICP, a serious obstetric complication, to be about 6-10 times higher than that of normal pregnancy (22). In our study, the gestational age (delivery) of pregnant women with ICP was significantly lower than that of healthy pregnant controls (**Table 1**), suggesting an increased probability of preterm birth in ICP patients. As such, early detection of and intervention in the setting of ICP effectively reduces both morbidity of and economic pressure on pregnant women and their infants. Unfortunately, clinical monitoring of ICP currently mainly depends on screening of biochemical indices such as TBA levels; such modalities have low sensitivity and specificity (7). It is thus necessary to investigate potential early sensitive molecular events in the setting of ICP and screen for sensitive biomarkers. Chen et al. (23) have reported MMP-2 and MMP-9 serum levels exhibited a progressive and significant elevation in mild and severe ICP patients compared with healthy pregnant women, indicating MMP-2 and MMP-9 could be reliably used as laboratory abnormalities for accurate diagnosis and sensitive grading of ICP in Chinese population. Jelski et al. (24) have reported the total ADH activity and class I ADH isoenzymes was significantly higher in women with ICP than in healthy pregnant, indicating that the activity of class I ADH isoenzymes may have a diagnostic significance. However, there are few studies on biomarkers for simultaneous diagnosis and prediction of ICP.

Multi-omics analysis is valuable in this context. There is a close relationship between proteome and metabolome in that the protein (e.g., enzyme) regulates metabolite concentrations. On the other hand, metabolites may affect protein abundance (25, 26). Proteomics and metabolomics have been widely used in mechanistic studies and in screening for clinical biomarkers of gynecological and obstetric conditions. Kosova et al. (27) noted significantly increased levels of various proteins related to preterm birth which may allow for development of a test for early preterm birth detection and resultant early intervention. Metabolomics researchers in Brazil and Auckland sought to identify, quantify, and validate potential metabolite predictors associated with preterm birth by utilizing metabolomics technologies (28). Hsu et al. (29) applied MS to identify 10 proteins with roles in the detection of preeclampsia, including α1-antitrypsin, α1-microglobulin, clusterin, and haptoglobin. Kim et al. (30) identified potential predictive biomarkers for gestational diabetes by analyzing proteomic profiles generated by SELDI-TOF MS; three peaks were noted to be significant.

In our previous study, a total of 38 differentially expressed proteins were identified; of them 29 were found to be upregulated and 9 were found to be downregulated in placental samples obtained from pregnant women with ICP. Bioinformatic analysis revealed most of the proteins to be functionally related to specific cellular processes, including fatty acid metabolism, apoptosis, and oxidative stress (12). In this continuing study, four metabolites were found to be upregulated and five to be downregulated in ICP patients as compared to controls (**Table 2**). Hierarchical cluster analysis likewise revealed significantly altered metabolite expression patterns between pregnant women with ICP and healthy controls (**Figure 1B**). Our metabolite pathway analysis findings were consistent with the results of proteomics pathway analysis; fatty acid metabolism was found to be significantly altered via both methods (**Table 3**). At the same time, we found the differentially expressed protein ACOX1 and the differentially expressed metabolite L-palmitoylcarnitine to both be involved in fatty acid metabolism (**Figure 1C**). Pearson correlations between ACOX1 and L-palmitoylcarnitine levels revealed levels of L-palmitoylcarnitine to be positively correlated with ACOX1 levels, indicating the presence of a regulatory relationship among these two compounds (**Figure 6D**). At the same time, there are few studies on the diagnostic and predictive value of ICP by ACOX1 and L-palmitoylcarnitine. In addition, metabolite pathway analysis revealed primary bile acid biosynthesis to be altered. Glycocholic acid, the most significant metabolite, was found to be increased in ICP patients, a finding consistent with the pathogenesis of ICP.

To validate our proteomic and metabolomic findings and further investigate the importance of identified proteins and metabolites, L-palmitoylcarnitine, glycocholic acid, and ACOX1 obtained from placentas of women with ICP were selected for further analysis. Findings confirmed L-palmitoylcarnitine, glycocholic acid, and ACOX1 to be significantly upregulated among ICP patients as compared to controls, thus confirming LC-MS/MS and ultra-performance LC-MS/MS data (**Figures 2** and **3A**). In order to further verify whether metabolites and proteins over-expressed in the placenta of pregnant women with ICP were secreted into serum, expression of serum levels of differential proteins and metabolites were validated in both groups. Results revealed serum levels of L-palmitoylcarnitine, glycocholic acid, and ACOX1 to be significantly elevated in ICP patients as compared to controls (**Figures 3B**–**D**) and levels of all three biomarkers were found to be positively associated with TBA levels (**Figures 6A–C**). At the same time, we found that serum levels of all three biomarkers to be significantly elevated in severe group as compared to mild group of ICP patients (**Figures 3E**–**G**), indicating that the three biomarkers may correlated with the severity of ICP.

L-palmitoylcarnitine, a long chain fatty acid derivative, is closely related to β-oxidation. L-palmitoylcarnitine levels affect the function of various enzymes, carrier proteins, and mitochondria (31). Levitsky et al. (32) demonstrated that L-palmitoylcarnitine, when transported into isolated rat liver mitochondria, induce swelling of the mitochondrial matrix. Bouchouirab et al. (33) found that serum levels of L-palmitoylcarnitine are significantly elevated in patients with type 2 diabetes mellitus. Ljubkovic et al. (34) found that diabetics have a significantly decreased rate of mitochondrial respiration fueled by L-palmitoylcarnitine that correlates with blood glucose dysregulation. Here, we found L-palmitoylcarnitine expression to be significantly increased in the placentas of ICP patients (**Figure 3A**), suggesting that overexpression of L-palmitoylcarnitine plays a key role in the disruption of placental fatty acid oxidation in the setting of ICP. We also found serum levels of L-palmitoylcarnitine to be significantly increased among ICP patients (**Figure 3B**). The AUC of L-palmitoylcarnitine was 0.896 (**Table 4**), indicating that this metabolite may be used as a diagnostic biomarker for ICP. At the same time, we found serum levels of L-palmitoylcarnitine among ICP patients to be significantly increased in the first and second trimesters (**Figures 4A, D**), underscoring an early metabolic change in ICP. However, the AUCs of L-palmitoylcarnitine were 0.657 and 0.727 (**Table 5**) in the first and second trimesters, respectively, indicating that the predictive value of this single metabolite has limitations.

Fatty acid β-oxidation occurs as a cyclical series of reactions and results in the progressive shortening of fatty acids (35). Each round of shortening generates reduced nicotinamide adenine dinucleotide, flavin adenine dinucleotide (hydroquinone form), and acetyl-coenzyme A (36). ACOX1 is the first and rate-limiting enzyme in the β-oxidation of very-long-chain fatty acids and also a major producer of hydrogen peroxide (37). Plubell et al. (38) reported that a high-fat diet induces ACOX1 overexpression in mouse epididymal adipose tissue. Shen et al. (36) reported a specific vulnerability of BRAFi/MEKi-persistent melanoma cells resulting from their predominant dependency on ACOX1-mediated peroxisomal fatty acid β-oxidation that can potentially translate into therapeutic opportunities. Ferdinandusse et al. (39) found that newborns with ACOX1 deficiency first exhibit hypotonia and seizures, and later a gradual loss of learning and speaking skills, usually starting between the ages of one and three. To date, there have been few studies concerning ACOX1 and the pathogenesis of pregnancy complications. In our study, overexpression of both serum and placental ACOX1 was observed among ICP patients (**Figures 2** and **3D**). The AUC of ACOX1 was 0.823, indicating that this protein may be used as a diagnostic biomarker for ICP (**Table 4**). Levels of serum ACOX1 among ICP patients were found to also be significantly increased in the first and second trimesters (**Figures 4C, F**), indicating that metabolism involving L-palmitoylcarnitine and ACOX1 undergoes early changes in ICP. The AUC of ACOX1 was 0.726 and 0.718 for the first and second trimesters (**Table 5**), respectively, indicating that this protein alone possesses a certain predictive value.

Bile acids, the end products of hepatic cholesterol metabolism, are formed in the liver, conjugated mainly with glycine and taurine and then secreted into bile (40). The pathophysiological basis of ICP is hypercholic acidemia. Both animal and *in vitro* experiments have confirmed hypercholic acidemia capable of inducing apoptosis of placental cells; placental apoptosis is significantly reduced after treatment with ursodeoxycholic acid (41–43). Clinical studies have also confirmed that the risk of fetal adverse complications is closely related to maternal glycocholic acid levels, with their incidence increasing significantly with the increase in maternal glycocholic acid levels (44). Treatment with ursodeoxycholic acid significantly reduces the incidence of adverse fetal complications (45, 46). Here, women with ICP were found to have significantly elevated levels of both serum and placental glycocholic acid as compared to healthy controls (**Figures 3A, C**). Brites et al. (47) have reported glycine/taurine bile acid ratio bellow 1.0 or glycocholic acid concentration above 2.0 μmol l−1 may be helpful in the diagnosis of ICP, our findings are in agreement with the study. The AUC of glycocholic acid was 0.985, indicating that this bile acid has a very high diagnostic value (**Table 4**). However, there are few studies on the predictive value of ICP by glycocholic acid. We found that serum levels of glycocholic acid among ICP patients were also significantly increased in the first and second trimesters (**Figures 4B, E**). However, the AUCs of glycocholic acid were 0.686 and 0.670 in the first and second trimesters (**Table 5**), respectively, indicating that the predictive value of this metabolite alone has certain limitations. Therefore, in this study, we conducted further analysis by combining the metabolites and proteins.

At the same time, this study analyzed the correlation between gestational age (delivery) and all three biomarkers. The results showed that the levels of gestational age (delivery) were negatively associated with all three biomarkers (**Figures 6E**–**G**), suggesting that the higher the level of these biomarkers, the more severe the disease, and the greater the probability of premature birth.

## Conclusion

In conclusion, these three biomarkers have good diagnostic value, whether applied separately or utilized together as a single indicator. The diagnostic value of glycocholic acid was the highest, up to 0.985. Multiple logistic regression analyses revealed that when levels of the three biomarkers were combined, the AUC increased to 0.993, indicating that the combined biomarker levels afforded more reliable ICP diagnosis than did the individual levels. At the same time, we found that the individual predictive value of these three biomarkers in the first and second trimester of pregnancy was not high. ACOX1, with AUCs of 0.726 and 0.718 in the first and second trimesters, respectively, was found to have the greatest predictive value. For L-palmitoylcarnitine, the predictive value in the second trimester was higher than that of early pregnancy. Although the diagnostic value of glycocholic acid was very high, its predictive value was limited, at only 0.686 and 0.670 in the first and second trimesters, respectively. Multiple logistic regression analyses revealed that when the levels of these three biomarkers were combined, the AUC increased to 0.891 and 0.932 in the first and second trimesters, respectively, indicating that the combined biomarker levels afforded more reliable ICP prediction than did the individual levels. We identified three differentially expressed proteins and metabolites in the serum of ICP patients and found that L-palmitoylcarnitine, ACOX1, and glycocholic acid together may serve as a new biomarker set for ICP diagnosis and prediction.

However, this is a preliminary work, the limitation of our study is the sample size studied. In the following study, we are prospectively recruiting and following additional ICP patients in order to validate our results. At the same time, the detection of these three biomarkers requires two different assays, which leads to some limitations in clinical application, the possible clinical applications require further investigation and optimization.

## Data Availability Statement

The datasets presented in this study can be found in online repositories. The names of the repository/repositories and accession number(s) can be found in the article/**Supplementary Material**.

## Ethics Statement

The studies involving human participants were reviewed and approved by the Institutional Review Board of Nanjing Medical University, and all participants provided written, informed consent (Nanjing Medical University Ethical review (2016) 241 number). The patients/participants provided their written informed consent to participate in this study.

## Author Contributions

TZ: Conceptualization, Methodology, Funding acquisition, Supervision. LL: Software, Project administration. RD: Writing - Review & Editing. NY: Writing - Original Draft. SZ: Writing - Original Draft. GW: Validation. YZ: Data Curation. TW: Investigation. PZ: Validation. JW: Resources. TY: Formal analysis. MC: Data Curation. CZ: Formal analysis. All authors contributed to the article and approved the submitted version.

## Funding

This study was supported by the National Natural Science Foundation of China (grant no.82171674 and 81671489), the Major Research Foundation of Jiangsu Science and Technology Department (grant no. BE2017628), Jiangsu Provincial Medical Youth Talent of the Project of Invigorating Health Care through Science, Technology and Education (QNRC2016167), the Foundation of Six Talent Peaks Project of Jiangsu Province (grant no. 2014-WSW-059 and WSN-184), Wuxi City Key Medical Talent of the Project of Invigorating Health Care through Science, Technology and Education (Z201601). Wuxi City Health Committee top-notch talent (BJ2020077), Jiangsu Province Health Committee “ Six One Project “ Top Talent Project (LGY2020023), Wuxi City Health Committee Youth Project (Q202032).

## Conflict of Interest

The authors declare that the research was conducted in the absence of any commercial or financial relationships that could be construed as a potential conflict of interest.

## Publisher’s Note

All claims expressed in this article are solely those of the authors and do not necessarily represent those of their affiliated organizations, or those of the publisher, the editors and the reviewers. Any product that may be evaluated in this article, or claim that may be made by its manufacturer, is not guaranteed or endorsed by the publisher.

## Abbreviations

ICP, Intrahepatic cholestasis of pregnancy; ACOX1, acyl-CoA oxidase 1; TBA, total bile acid; LC-MS/MS, liquid chromatography-tandem mass spectrometry; ALT, alanine transaminase; AST, aspartate transaminase; VIP, variable important for the projection; HELLP, elevated liver enzymes and low platelets; BSA, bovine serum albumin; IPI, International Protein Index; HESI, heated electrospray ionization; AU, arbitrary units; TBS, Tris-buffered saline; PBS, phosphate buffered saline; ROC, Receiver operator characteristic; CIs, confidence intervals; AUCs, referencing areas under the curves; SEs, means ± standard errors.
