# Supplementary material for: Studies on Novel Diagnostic and Predictive Biomarkers of Intrahepatic Cholestasis of Pregnancy Through Metabolomics and Proteomics
Source: Front Immunol. 2021 Oct 14;12:733225. doi: 10.3389/fimmu.2021.733225 (PMC8552060; doi:10.3389/fimmu.2021.733225)

Figure A is the western blot original result of ACOX1 protein expression in placental tissue, according to the Anti-ACOX1 [Abcam, EPR19038] specification, there are two bands (Figure D). Figure B is the western blot original result of actin protein (internal control) expression in placental tissue. Figure C is the original gel picture.


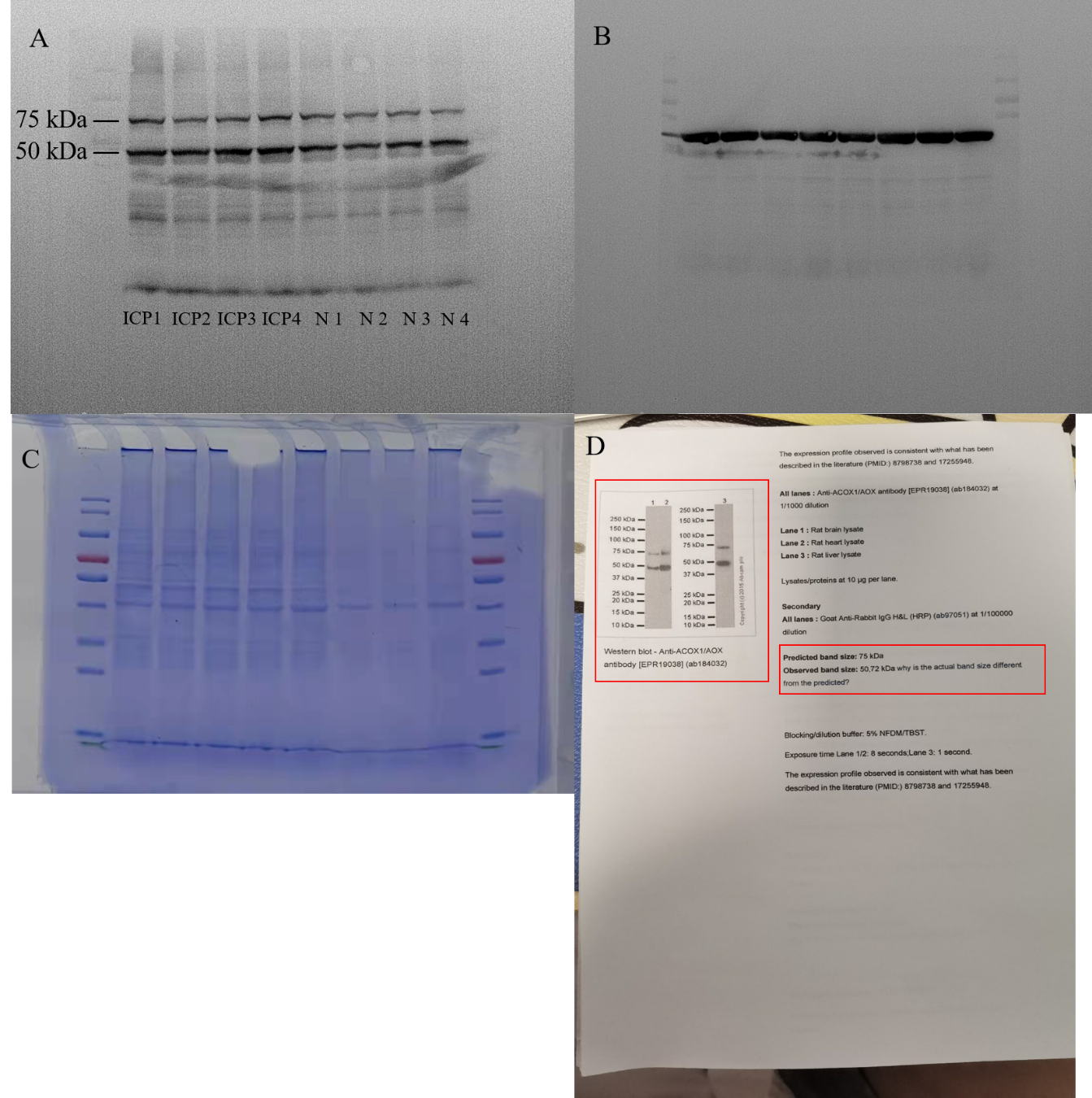

Supplement: Supplementary file 5 [file DataSheet_5.docx]
